# Fluorescent and Colorimetric Electrospun Nanofibers for Heavy-Metal Sensing

**DOI:** 10.3390/bios7040061

**Published:** 2017-12-15

**Authors:** Idelma A. A. Terra, Luiza A. Mercante, Rafaela S. Andre, Daniel S. Correa

**Affiliations:** 1 Nanotechnology National Laboratory for Agriculture (LNNA), Embrapa Instrumentação, São Carlos 13560-970, SP, Brazil; iaterra@yahoo.com.br (I.A.A.T); lela_rsa@hotmail.com (R.S.A.); 2 PPG-CEM, Department of Materials Engineering, Center for Exact Sciences and Technology, Federal University of São Carlos (UFSCar), São Carlos 13565-905, SP, Brazil; lamercante@gmail.com (L.A.M.); 3 PPGQ, Department of Chemistry, Center for Exact Sciences and Technology, Federal University of São Carlos (UFSCar), São Carlos 13565-905, SP, Brazil

**Keywords:** electrospinning, optical sensors, heavy metals

## Abstract

The accumulation of heavy metals in the human body and/or in the environment can be highly deleterious for mankind, and currently, considerable efforts have been made to develop reliable and sensitive techniques for their detection. Among the detection methods, chemical sensors appear as a promising technology, with emphasis on systems employing optically active nanofibers. Such nanofibers can be obtained by the electrospinning technique, and further functionalized with optically active chromophores such as dyes, conjugated polymers, carbon-based nanomaterials and nanoparticles, in order to produce fluorescent and colorimetric nanofibers. In this review we survey recent investigations reporting the use of optically active electrospun nanofibers in sensors aiming at the specific detection of heavy metals using colorimetry and fluorescence methods. The examples given in this review article provide sufficient evidence of the potential of optically electrospun nanofibers as a valid approach to fabricate highly selective and sensitive optical sensors for fast and low-cost detection of heavy metals.

## 1. Introduction

The accelerated deterioration of the environment caused by pollutants produced due to rapid industrialization and urbanization has become a serious global challenge that is predicted to become worse in the future [1,2,3,4,5]. In this scenario, heavy metals appear as pollutants of immense biological and environmental concern as they are nondegradable and tend to accumulate in ecological systems and in the food chain, causing serious problems for the environment and human health [4,5,6,7,8]. The accumulation of such metals in the human body can cause severe damage to mucus tissue, the intestinal tract, the skeletal system, the central nervous system, the liver, kidneys and reproductive systems [3,4,5,7].

Maximum permissible levels of heavy metal ions in the environment have been recommended by several environmental agencies including the World Health Organization (WHO), US Environmental Protection Agency (EPA) and European Medical Agency (EMA) [7,9]. In general, most of these permissible limits fall in the range from ppt to ppm levels [3,5]. Therefore, the development of rapid and inexpensive strategies for sensing heavy metals for the early detection of pollution in living systems and in the environment becomes highly desirable. 

Traditional analytical methods such as atomic absorption spectroscopy (AAS), inductively coupled plasma–mass spectrometry (ICP–MS), inductively coupled plasma–optical emission spectroscopy (ICP–OES), mass spectrometry (MS) and X-ray fluorescence spectroscopy (XPS) can provide accurate quantitative information on metal ions, but their application can be hampered by expensive equipment, complex procedures and inflexibility for on-site analysis [4,10]. In light of these drawbacks, emphasis is currently being placed on the development of sensor devices that offer high sensitivity, short response times, and selectivity for the in-situ/on-site screening of metal ions. Potential candidates to meet these requirements include laser-induced breakdown spectroscopy (LIBS) [11], and electrochemical [12] and optical [13] techniques. Among them, optical sensors that allow on-site and real-time detection without the use of any complicated spectroscopic instrumentation have received great attention for the biosensing of different pollutant species [10,14,15,16,17]. 

The successful development of a sensible and robust optical chemical sensor is intrinsically related to the nature of the platform on which it is constructed [6,18,19]. With recent advances in nanomaterials, new strategies are emerging to design novel optical biosensor platforms. These materials can lead to significant improvement in the performance of sensors in terms of sensitivity, selectivity, multiplexed detection capability and portability [3,4]. To date, various nanomaterials, such as graphene and graphene oxide, quantum dots, carbon nanotubes, and metallic and magnetic nanomaterials, have been successfully exploited for designing sensors relying on different optical signal transduction systems [4,15]. 

Compared with other nanomaterials, nanofibers obtained by electrospinning can be applied in optical sensors with several advantages including easy fabrication and functionalization, low cost, easy detection, and customized properties, such as chemical composition, structure, morphology, porosity and diameter [14,15,16,20,21,22]. In addition, considering that the electrospun nanofibers (ESNFs) are obtained as membranes/mats and can be taken out after the detection process, optical sensors based on ESNFs do not contaminate the detection solution and allow easy post-treatment after the detection process [14,15,20]. 

**Figure 1:**
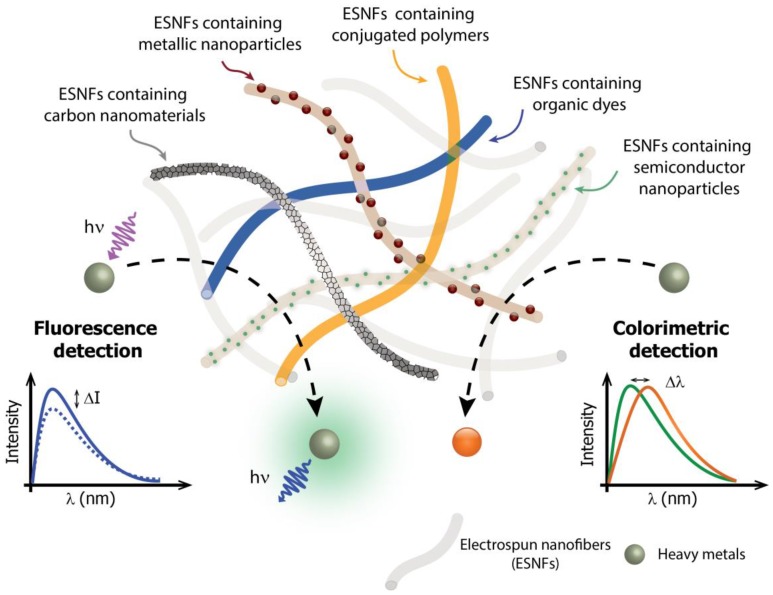
Scheme of electrospun nanofibers (ESNFs) modified by distinct nanomaterials for applications in optical sensors for heavy-metal detection. The lower part of the figure provides a schematic showing the principles of optical detection by the fluorescence quenching (left) or by the color change (right) of the ESNFs in the presence of the heavy metal ion.

The possibility to build up multifunctional nanostructures by functionalizing the nanofibers with a wide range of optically active materials, as illustrated in Figure 1, allows the electrospinning technique to design optical biosensors with improved performance [20]. Optically active ESNFs can be obtained using three main approaches: (i) by embedding absorbing or luminescent chromophores or NPs into transparent and optically inert polymers; (ii) by using conjugated polymers, which can intrinsically absorb/emit light and (iii) by attaching optically active nanosystems to the polymer surface through suitable chemical functionalization [17].

The most common methods applied for the optical sensing of heavy metals using ESNFs are those based on light absorption or light emission. Absorption or colorimetric sensing is accomplished using an indicator that changes its color upon binding to the analyte; this change is not only spectroscopically determined but can also be visible. In light-emission methods, the analyte concentration is determined by the change in the emission properties of a fluorophore after being excited by a defined wavelength [23]. Therefore, this paper reviews the recent progress on the development of ESNF-based optical sensors using colorimetric or fluorescent methods for the determination of heavy metals. Here we intend to give the readers a detailed evaluation on the distinct optically active electrospun nanofibers based approaches employed for the analysis of some hazardous metal ions (i.e., Fe(III), Hg(II), Cu(II), Ni(II), Cr(III) and Pb(II)). The prominent examples, reported in the last five years, of fluorescent and colorimetric electrospun nanofibers used for heavy-metal detection, are summarized in Table 1 and Table 2. Firstly, we present some examples of optical sensors based on fluorescent ESNFs, and then, colorimetric detection of heavy metals based on ESNFs functionalized with organic molecules, nanoparticles and conjugated polymers are overviewed. Finally, the review indicates trends and directions regarding the latest advances in the development of electrospun nanofibers for optical detection of hazardous ions.

## 2. Electrospun Nanofiber-Based Optical Sensors for Heavy-Metal Detection

### 2.1. Optical Detection Using Fluorescence

Luminescence is the phenomenon of light emission by a substance that was not strongly heated, where the emission is not associated with thermal radiation. Depending on the nature of the excited state and emission rates (lifetime), luminescence is divided into two categories, namely fluorescence and phosphorescence. The theoretical background of some of the main optical mechanisms employed in optical sensors, such as fluorescence quenching, Stokes shift and resonance energy transfer (RET), are fundamental concepts that allow the analysis of different properties of materials [31,51,52,53]. The high sensitivity provided by fluorescence is an important feature for sensing materials applied in optical sensors for varied applications. 

**Figure 2:**
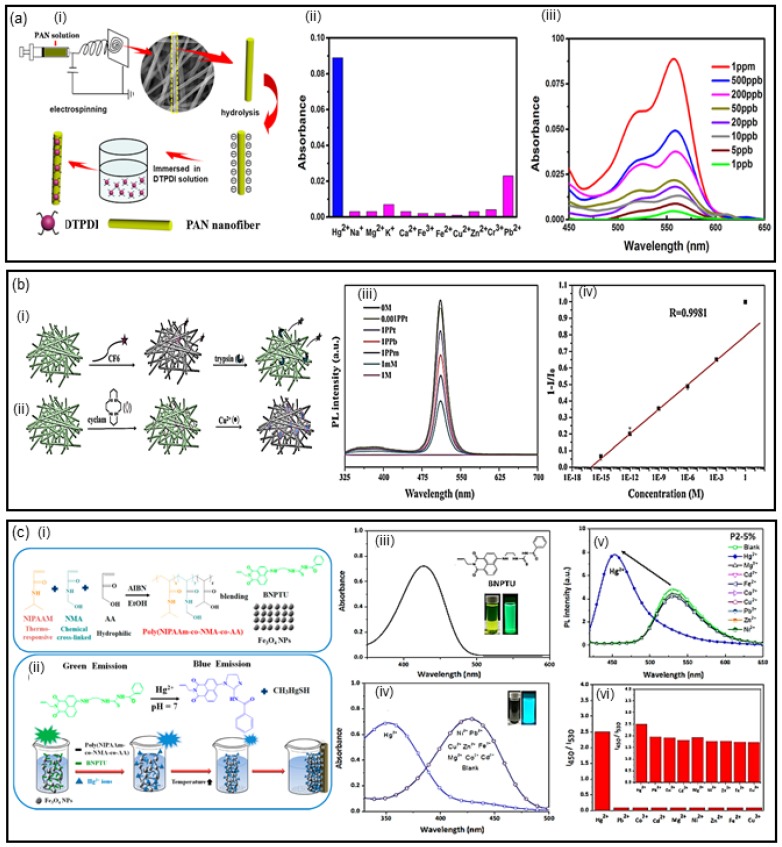

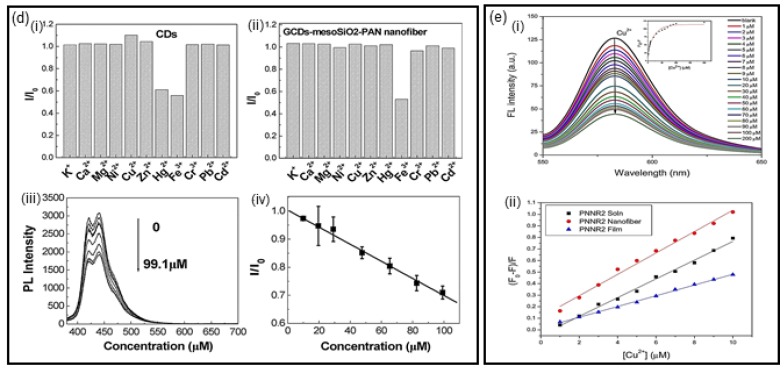
(**a**) (i) Schematic representation for the fabrication of fluorescent nanofibrous membrane (NFM), (ii) absorbance of FNFM after being immersed into the aqueous solution of metal ions and (iii) UV–vis absorption spectra in the presence of Hg(II) with various concentrations. Adapted and reprinted with permission from [27]. Copyright 2017 Elsevier. (**b**) Schematic illustration of (i) trypsin and (ii) Cu(II) sensing based on the CPBQD/PMMA (CsPbBr_3_ perovskite quantum dots/polymethylmethacrylate), (iii) Photoluminescence (PL) spectra of the CPBQD/PMMA in an aqueous medium of different Cu(II) concentrations and (iv) relationship between the PL intensity and Cu(II) concentration. Reprinted with permission from [32]. Copyright 2017 Royal Society of Chemistry. (**c**) (i and ii) Schematic illustration of multifunctional sensory electrospun nanofibers (ESNFs) synthesized from poly(NIPAAm-*co*-NMA-*co*-AA), 1-benzoyl-3-[2-(2-allyl-1,3-dioxo-2,3-dihydro-1Hbenzo[de]isoquinolin-6-amino)-ethyl]-thiourea (BNPTU) and Fe_3_O_4_ blends with magnetic fluorescence emission, (iii) absorption spectra, (iv) variation of absorption spectra for different metal ions, (v) variation in the normalized PL spectra for different metal ions and (vi) fluorometric response ESNFs. Reprinted with permission from [28]. Copyright 2017 MDPI. (**d**) (i) Schematic illustration of the selectivity of carbon quantum dot (CD), (ii) CD/mesoSiO_2_/PAN nanofibers for Fe(III) detection, (iii) PL response and (iv) linear fit of the PL intensity towards Fe(III) of the CD/mesoSiO_2_/PAN nanofibers. Reprinted with permission from [29]. Copyright 2016 Springer. (**e**) (i) Fluorescence spectra of PNNR2 solution in methanol/Tris–HCl for different concentrations of Cu(II) ions and (ii) Stern–Volmer plots of PNNR2 in different states for Cu(II) detection. Adapted and reprinted with permission from [33]. Copyright 2016 Springer.

In this topic, we discuss some of the most recent work that employed electrospun nanofibers for different heavy-metal sensing through optical detection using fluorescence. In recent work, Ma and coworkers [27] reported the fabrication of fluorescent nanofibrous membrane (NFM) using the electrospinning technique. The NFMs were obtained through immobilization of dithioacetal-functionalized perylenediimide (DTPDI) on hydrolyzed polyacrylonitrile (PAN) nanofiber membranes, as illustrated in Figure 2a (i). When the NFMs were immersed in Hg(II) aqueous solution, the reaction of the DTPDI with Hg(II) takes place, due to the sulfur–mercury affinity. Consequently, the C–S–C bonds of DTPDI were broken to produce hydrolysate, which was adsorbed on DTPDI through π–π stacking. The selectivity of NFM was analyzed with K(I), Na(I), Mg(II), Ca(II), Cu(II), Zn(II), Fe(II), Fe(III) and Cr(III) ions (Figure 2a (ii)). The high sensitivity and selectivity toward Hg(II) were observed in the absorbance measured of the NFMs, where there is an increase of intensity as a function of Hg concentration, with a detection limit of 1.0 × 10^−3^ mg·L^−1^ for Hg(II) in water (Figure 2a (iii)). The study demonstrated that the sensing of NFMs with Hg(II) was reversible and stable, while the selectivity of chemosensing was evaluated for different heavy metal ions, with no relevant interference. 

Electrospun fiber membranes as multifunctional sensors for biomolecules, metal ions and pH have attracted attention. In this direction, Wang et al. [32] developed multifunctional ESNFs based on CsPbBr_3_ perovskite quantum dots (CPBQDs) encapsulated into polymethylmethacrylate (PMMA) electrospun nanofibers (CPBQD/PMMA). The detection of trypsin, Cu(II) and pH was based on the efficient fluorescence resonance energy transfer (FRET) process occurring between CPBQD/PMMA ESNFs and Rhodamine 6G (R6G) or cyclam, respectively, due to the high quantum yield (88%) of CPBQD/PMMA ESNFs (Figure 2b (i) and (ii)). Such a feature enables the high-efficiency FRET process between the CPBQD/PMMA and Cu(II) after attaching cyclam, which is used as the probe for Cu(II). The detection of Cu(II) by CPBQD/PMMA FM (fibermembrane)–cyclam is measured by quenching PL (Figure 2b (iii)), which occurs due to the absorption band of cyclam–Cu(II) at 520 nm matching the emission band of the CPBQD/PMMA FM. This behavior leads to a detection limit of 6.0 × 10^−11^ mg·L^−1^ for Cu(II) in aqueous medium, highly suitable for real sample analysis. The selectivity test showed that the CPBQD/PMMA–cyclam is specific to Cu(II) compared with the same concentration of Zn(II), Mn(II), Cr(II), Fe(II), Co(II), Na(I) and K(I) ions, due to the low-efficiency FRET processes between the CPBQD/PMMA and these ions. 

Another multifunctional fluorescent ESNF was reported by Liang et al. [28], obtained by the combination of poly (*N*-isopropylacrylamide)-*co*-(*N*-methylolacrylamide)-*co*-(acrylic acid), the fluorescent probe 1-benzoyl-3-[2-(2-allyl-1,3-dioxo-2,3-dihydro-1Hbenzo[de]isoquinolin-6-amino)-ethyl]-thiourea (BNPTU) and magnetite nanoparticles (NPs), as displayed in Figure 2c (i and ii). The produced NFMs exhibited high sensitivity toward magnetism, temperature and mercury ions (Hg(II)). The limit of detection of 2.0 × 10^−2^ mg·L^−1^ for Hg(II) in aqueous solution was obtained by absorbance and photoluminescence measurements, where a blue-shift of maximum emission was noted (Figure 2c (iii and v)). High selectivity and sensitivity for Hg(II) was observed compared to different metals (Figure 2c (iv and vi)). The same blue-shift was observed by fixing the Hg(II) concentration and varying the temperature between 30 and 60 °C. The blue-shift was observed due to the Hg(II) ion transforming the thiourea unit of BNPTU into an imidazoline moiety, which has weak electron-donating ability (Figure 2c (i)). On the other hand, a change in the absorption and photoluminescence (PL) peak for other metal ions was not observed (Figure 2 c (iv)), ensuring the selectivity for Hg(II) ion. The magnetic fluorescent ES nanofibers can be used as naked-eye sensors for accessible and practical applications as multifunctional sensing devices.

Li et al. [29] reported the development of carbon quantum dot (CD)-encapsulated mesoporous silica/polyacrylonitrile (CD/mesoSiO_2_/PAN) electrospun nanofibers. The use of CDs has been explored in various areas, owing to their excellent physical and chemical properties. By encapsulation of the CDs into the polymer nanofiber, the authors preserved the CDs’ sensory capabilities, improving sensibility and stability of material. CD/mesoSiO_2_/PANs are promising sensitive photoluminescence sensors of Fe(III). The study compared the sensing capabilities of CDs in solution and CD/mesoSiO_2_/PAN nanofibers, and the latter showed higher efficiency, photoluminescence stability and improved selectivity. In Figure 2d (i) and (ii) are displayed the selectivity of CDs in solution and CD/mesoSiO_2_/PAN nanofibers for Fe(III) detection, respectively. The selectivity was ascribed to a coordinated interaction between Fe(III) and the phenol hydroxyl groups of CDs, that brought the photoluminescence quenching signal as a function of the Fe(III) concentration, (Figure 2d (iii)), resulting from the interaction of the metal ions with oxygen in the presence of the groups of the CDs. The limit of detection of Fe(III) in aqueous solution was determined as 0.2 mg·L^−1^ (Figure 2d (iv)). The incorporation of CDs on the electrospun nanofibers improved the luminescence properties of CDs, and qualified the CD/mesoSiO_2_/PAN nanofibers as a promising ‘turn-off’ and ‘label-free’ fluorescence sensor for heavy metals.

The enhanced sensitivity of electrospun nanofibers is, in general, attributed to their larger surface area compared to thin films. This improvement was confirmed by the study reported by Wu and coworkers [33], where a comparison between the properties of electrospun nanofibers versus dip-coating films of poly[(*N*-isopropylacrylamide)-*co*-(*N*-hydroxymethyl acrylamide)-*co*-(4-rhodamine hydrazonomethyl-3-hydroxy-phenyl methacrylate)] [poly(NIPAAm-*co*-NMA-*co*-RHPMA)-PNNR] copolymers was carried out, under the conditions of different molar ratios of NIPAAm, NMA and RHPMA monomers (PNNR1, PNNR2 and PNNR3). The latter showed a high selectivity and sensitive recognition of Cu(II) through fluorescence measurements (Figure 2e (i)). The increase of Cu(II) concentration led to a quenching of photoluminescence, which occurs due to the paramagnetic effect of the d9 system of the Cu (II) ion, which suggests a photoinduced electron transfer from singlet fluorophore (rhodamine) excited state to paramagnetic metal center. [33] The Stern–Volmer constants of PNNR copolymers in nanofibers and dip-coated films were obtained from the slopes showed in Figure 2e (ii), indicating that PNNR nanofibers have a much higher sensitivity as compared to films. The limit of detection of Cu(II) to PNNR nanofibers was determined as 6.4 × 10^−3^ mg·L^−1^. The study suggests the PNNR nanofibers have potential applications as “on–off” types of fluorescent sensors for Cu(II) ions, as well as for the application in pollutant separation and water purification.

### 2.3. Optical Detection Using Colorimetry

As previously mentioned, the colorimetric detection of a specific analyte induces changes in the absorption band of the material, resulting in color changes that can be easily interpreted by the naked eye. As a consequence, the detection response measurement does not require complex and expensive equipment, simplifying the sensing apparatus [8,54,55]. However, the color change can be highly influenced by experimental parameters such as luminosity and visualization angle. To eliminate misrecognition, colorimetric devices have the color change followed by absorbance measurements using a standard UV-vis spectrometer [34]. Therefore, the absorbance band shift can be precisely measured and the sensor response determined according to the type and concentration of the target analyte for more accurate systems. 

**Figure 3:**
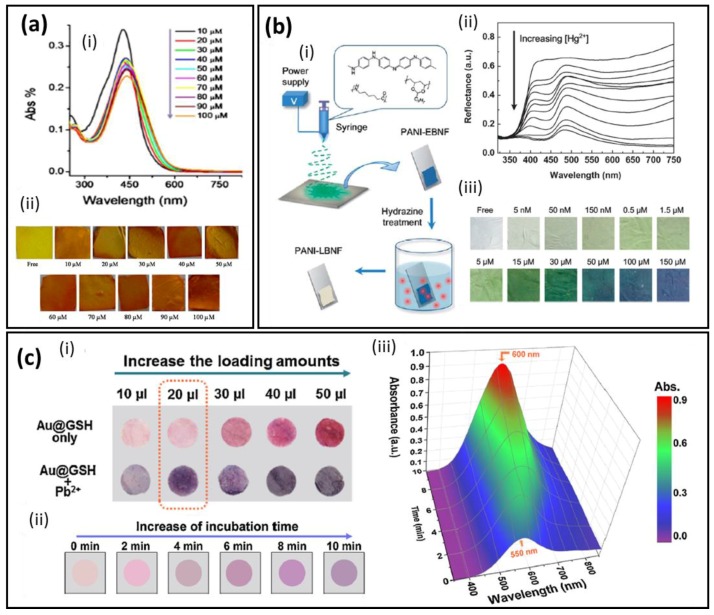
(**a**) (i) UV–vis for different concentrations of Pb(II) and (ii) nanofiber images showing a visual color change. Adapted and reprinted with permission from [45]. Copyright 2016 Elsevier. (**b**) (i) Schematic illustration of the preparation of PANI-LBNF sensing membranes by the combination of electrospinning and hydrazine treatment, (ii) reflectance spectra and (iii) optical colorimetric responses of the PANI-LBNF sensor strips after incubation in different Hg(II) concentrations. Adapted and reprinted with permission from [47]. Copyright 2014 Royal Society of Chemistry. (**c**) (i) Digital photo images of the loading optically active species amount and the corresponding samples after incubation with Pb(II), (ii) kinetic absorption response of the strips as a function of time and (iii) time-dependent visualization of CIE Lab color changes versus Pb(II) concentration. Adapted and reprinted with permission from [44]. Copyright 2014 Elsevier.

Organic dyes with conjugated aromatic systems have been largely employed for heavy metal detection [56,57]. The detection mechanism is pretty easy and involves the reprecipitation of the metal ions by a complexation process with the organic molecules. However, not many systems are based on NFM substrates yet, which indicates a whole world of possibility to be explored in the next years. Curcumin is one of the organic dyes employed for heavy metal detection and it is reported by Raj et al. [45] as a simple biocompatible and selective system for Pb(II) colorimetric detection. The strips were produced by electrospinning a composite mixture of cellulose acetate and curcumin, yielding curcumin-loaded cellulose acetate nanofibers (CC–CA). A colorimetric study was carried out at different pH levels for optimization of the operating conditions. The authors found out that below pH 4, the absorbance intensity changed without visual color alteration. From pH 5 to 11 represented better conditions without significant absorbance or color changes, and pH 5 was set as standard. The sensitivity was tested from 2.07 × 10^−3^ mg·L^−1^ to 207 mg·L^−1^ of Pb(II) ions and presented a linear decrease of the absorbance, a limit of detection of 4.1 mg·L^−1^ and a visual color change from yellow to orange, as displayed in Figure 3a. The color change is a result of the Pb(II) complexation by curcumin chelating. CC–CA strips proved to be selective for Pb(II) among eight different metal ions solutions (Ba(II), Ca(II), Co(II), Cd(II), Cu(II), Mg(II), Ni(II) and Zn(II)) with 1 mM of concentration. No color changes were observed on the CC–CA strips for ions distinct from Pb(II). The authors attributed the CC–CA strips’ selectivity to the strong and stable complex formed between curcumin and Pb(II) ions by the α,β-unsaturated β-diketo moiety. During the complexation, the enolic protons are replaced by the metallic ion, changing the electronic configuration and thus the materials’ color. Saithongdee et al. [49] also studied the performance of curcumin in zein NFM strips as chemosensor and the pH influence on the Fe(II) ion detection, with optical limit of detection of 0.4 mg·L^−1^ and linear visual color change with the Fe(II) ion concentration. Such results demonstrate the flexibility of the sensor’s selectivity for the detection of different heavy metal ions with the same optically active species by carefully choosing the associated material.

Dimethylglyoxime (DMG) is another organic aromatic molecule, which acts by complexing palladium and nickel ions. A colorimetric sensor for Ni(II) detection in tap water and laboratory waste water was fabricated based on polycaprolactam nanofibers combined with dimethylglyoxime in glass slides (N6-DMG@glass) by Najarzadekan et al. [48]. The strips presented a good performance with visual color change from colorless to red in the linear range from 5 × 10^−3^ to 1 × 10^−1^ mg·L^−1^ and a detection limit of 2 × 10^−3^ mg·L^−1^. However, selective tests showed significant interference for Cu(II) in concentrations higher than 6 × 10^−1^ mg·L^−1^ and higher than 1 mg·L^−1^ for seven other ions. 

Sensors fabricated with conjugated polymers have shown better performance with increased sensitivity when compared with other conjugated systems such as organic dyes. This behavior can be attributed to delocalized π-bonds in a long range which enable a faster charge transfer and an increased sensitivity. In the case of heavy-metal ion detection, conducting polymers will interact with the heavy metal ions by a redox mechanism, changing the conjugation of the bonds and consequently the material’s absorbance/reflectance. This change in the oxidation degree can also lead to heavy metal ion complexation. As is known, polyaniline (PANI) is a classic conjugated polymer that, besides modulation of its properties by the oxidation degree, has amine and imine groups, which can be protonated and deprotonated according to the amount of available H^+^, resulting in different colors varying from white to blue. For example, emeraldine base (EB) is the PANI half-oxidized state and it can be converted to the emeraldine salt according to the amine and imine group interactions or in leucoemeraldine base (LB) according to the variation of the oxidation degree. For sensors fabricated with PANI, besides the oxidation process, a second mechanism take place through amine and imine group protonation. The amine and imine groups will interact with the ions, changing the doping state, and consequently the optical properties. Si et al. [47] combined the direct fabrication of conjugated polymer nanofiber membranes followed by a functionalization step to convert the optically active species to a more interesting form. Thus, the authors showed the direct interaction between PANI and Hg(II) ions through the fabrication of a sensitive and selective sensor based on nanofiber membranes (PANI-LBNF), as illustrated in Figure 3b. Nanofiber membranes were obtained by the electrospinning technique followed by an emeraldine base reduction to leucoemeraldine base with hydrazine vapor, and they were used as probes for specific interactions with Hg(II) ions in aqueous medium. In the presence of Hg(II), PANI-LBNF undergoes by ether a protonation and oxidation process, showing a highly selective and sensitive response with a distinct color change from white to yellow/green, green and blue according to the Hg(II) concentration. The sensor response based on the color change could be characterized by the intensity variation of the reflectance bands at 440 and 645 nm. A 1.0 × 10^−3^ mg·L^−1^ concentration was the smallest concentration able to be detected with the naked eye, and was set as the sensor detection limit. The authors also used a novel colorimetric framework that can be compared to human perception to confirm the membrane color change along with the Hg(II) concentration, confirming the reliability of the PANI use for this type of detection. 

Regarding inorganic materials for colorimetric applications, metallic nanoparticles are very interesting due to their size-dependent optical properties [58]. Gold nanoparticles (AuNPs) are optically active materials that have attracted great attention for heavy metal detection [59,60,61,62,63,64]. For instance, Li et al. [44] reported for the first time the use of nanofiber membranes as a substrate for AuNPs as optically active sensors (Figure 3c). Furthermore, the authors employed a complex functionalization with immobilized *L*-glutathione (GSH) along with AuNPs (Au@GSH) on the surface of nylon 6/polyvinylidene-fluoride NFM for Pb(II) colorimetric detection. The GSH was added with the purpose of a chelating agent and also to stabilize the AuNPs through the thiol group, forming a covalent Au–S bond. The NFM strips decorated with Au@GSH presented a vibrant pink color due to the well-dispersed Au@GSH functionalization. Upon addition of Pb(II) aqueous solution, the strips’ color changed from pink to purple due to the distance decrease among Au@GSH, as a result of the Pb(II) ion complexation with Au@GSH. The sensor proved to be highly sensitive even by the naked eye, with a recognition limit of 1.0 × 10^−1^ mg·L^−1^. The sensor selectivity was tested against several salts, proteins and 10 metal cations, showing no apparent response changes, proving the high specificity toward Pb(II). The selectivity was attributed to factors such as the strong ability of Pb(II) binding with glutathione carboxylate ions, and also, that the –SH groups are occupied by Au nanoparticles preventing interactions with Hg(II) ions, and the –NH_2_ groups are protonated due to the water pH used, preventing or at least decreasing considerably the binding interactions with ions such as Zn(II), F(II) and Cd(II). A colorimetric framework for quantitative analysis and gradient sensitivity was also carried out, confirming the color changes for a wide range of Pb(II) concentrations (Figure 3b (iii)). The NFM chromic strips showed a performance 28-fold better than the paper-based one.

## 3. Conclusions and Future Outlook

Recent investigations reporting the use of electrospinning techniques to produce fluorescent and colorimetric electrospun nanofibers aiming at the specific detection of heavy metals were surveyed in this review article, where remarkable results for the analysis of both artificial and real samples were demonstrated. Electrospinning allows the production of nanofibers with high specific surface area, varied morphology and diameter, and the possibility of chemical functionalization. For instance, optically active ESNFs can be functionalized with metal nanoparticles, conjugated polymers and carbon-based nanomaterials, aiming at improving the sensor performance in terms of sensitivity and limit of detection towards heavy metal detection. The physicochemical properties of the nanomaterial and the polymeric matrix can be independently tailored and integrated into a single body to achieve an adaptable functionalization in order to obtain a high selectivity and sensitivity towards a specific heavy metal ion.

Among the several materials employed for producing optically active ESNFs, metallic and semiconducting nanoparticles appear to be the most promising, owing to the wide available chemical routes for their syntheses, facile surface chemistry, broad optical/electrical properties and low cost of production. Colorimetry and fluorescence are outstanding optical transduction mechanisms once the nanofiber membranes can have their composition modulated, according to the analyte investigated, in order to fabricate highly selective and sensitive portable sensors, enabling fast and naked-eye visual interpretation. Although electrospinning has started to move from academia to industry, some bottlenecks and pitfalls need to be solved for large-scale applications, which include: increasing the production rate of electrospun nanofibers, improving the control of processing parameters and surface modification methods, and enhancing sensor stability and limits of detection for a wide range of heavy metals. In spite of that, colorimetric and fluorescent ESNF sensors are shown to be highly suitable for real sample applications regarding heavy metal detection, and it is believed that the applications of such nanofibers in the sensors area will continue to grow in the next years.

## Figures and Tables

**Table 1:** Recent representative examples reported in the last five years of fluorescent-based electrospun nanofibers (ESNFs) for heavy-metal detection.

Analyte	Polymeric matrix	Recognition material	LOD (mg·L^−1^)	Reference
***Single detection***				
*Hg(II)*	PCL	AuNC	5.0 × 10^−8^	[24]
	EC	EMIMBF4	1.4 × 10^−5^	[25]
	poly(MMA-*co*-BNPTU-*co*-RhBAM)	BNPTU	4.0 × 10^−3^	[26]
	PAN	DTPDI	1.0 × 10^−3^	[27]
	poly(NIPAAm-*co*-NMA-*co*-AA)	BNPTU	2.0 × 10^−2^	[28]
*Fe(III)*	PAN	CDs	0.2	[29]
	poly(HEMA-*co*-NMA-*co*-NBD)	SRhBOH	5.6	[30]
*Ni(II)*	PAN-PAA	PAN-PAA	7.0 × 10^−3^	[31]
*Cu(II)*	PMMA	CsPbBr_3_ QDs	6.0 × 10^−11^	[32]
	PNNR	PNNR	6.4 × 10^−3^	[33]
	CA	DTT.AuNC	5.0 × 10^−2^	[34]
	poly(MMA-*co*-AHPA)	RhB-hydrazine	9.5 × 10^−2^	[35]
	poly(NIPAAm-*co*-NMA)	F-phen	-	[36]
*Al(III)*	PU	R2PP	2.0 × 10^−4^	[37]
***Multiple detection***				
*Cu(II)/Cr(III)*	CA	1,4-DHAQ	2.0 × 10^−4^ for Cu(II) and Cr(III)	[38]
*Co(II)/Zn(II)*	PU	DS-5N, FL-5N and NBD-5N	2.0 for Co(II) and 3.0 for Zn(II)	[39]
*Fe(III)/Cr(III)/Hg(II)*	PVA	SRD and SSRD	5.6 × 10^−2^ for Fe(III), 5.2 × 10^−2^ for Cr(III) and 0.1 for Hg(II)	[40]
*Pb(II)/Hg(II)/Fe(III)/Mn(II)/Ni(II)/Cd(II)*	PAN	CPEs	-	[41]

poly(NIPAAm-*co*-NMA-*co*-AA)—poly(*N*-isopropylacrylamide)-*co*-(*N*-methylolacrylamide)-*co*-(acrylic acid), BNPTU—1-benzoyl-3-[2-(2-allyl-1,3-dioxo-2,3-dihydro-1Hbenzo[de]isoquinolin-6-ylamino)-ethyl]-thiourea, poly(MMA-*co*-BNPTU-*co*-RhBAM)—(poly(methylmethacrylatete-*co*-1,8-naphthalimide derivatives-*co*-rhodamine derivative), EC—ethyl cellulose, EMIMBF4—1,2-bis(4-methoxybenzylidene)hydrazine, PCL—polycaprolactone, AuNC—gold nanoclusters, PAN—polyacrylonitrile, DTPDI—dithioacetal-modified perylenediimide, SRhBOH—spirolactam rhodamine derivative, CDs—carbon dots, PAN-PAA—pyridylazo-2-naphthol-poly(acrylic acid), poly(MMA-*co*-AHPA)-poly(methylmetacrylate-*co*-4-aldehyde-3-hydroxyphenylacrylate), RhB—rhodamine, CA—cellulose acetate, DTT.AuNC—dithiothreitol-capped gold nanoclusters, F-phen—1,10-phenanthroline, PMMA—poly(methylmetacrylate), QDs—quantum dots, PNNR—poly[(*N*-isopropylacrylamide)-*co*-(*N*-hydroxymethylacrylamide)-*co*-(4-rhodamine hydrazonomethyl-3-hydroxy-phenylmethacrylate), PU—polyurethane, R2PP—rhodamine-based probe, 1,4-DHAQ—1,4-dihydroxyanthraquinone, DS-5N—dansylchloride-tetraethylenepentaamine, FL-5N—fluorescamine-tetraethylenepentaamine, NBD-5N—7-chloro-4-nitrobenz-2-oxa-1,3-diazole-tetraethylenepentaamine, PVA—poly(vinyl alcohol), CPEs—conjugated polyelectrolytes.

**Table 2:** Recent representative examples reported in the last five years of colorimetric-based ESNFs for heavy-metal detection.

Analyte	Polymeric Matrix	Recognition Material	LOD (mg·L^−1^)	Reference
*Pb(II)*	CA	PMDA	1.0 × 10^−2^	[42]
	PAN	PCDA and PCDA-5EG	1.0 × 10^−1^	[43]
	PA6/PVDF	Au@GSH NPs	1.0 × 10^−1^	[44]
	CA	curcumin	4.1	[45]
*Hg(II)*	PVA	AuNC	1.0 × 10^−3^	[46]
	PA6/PVB	PANI	1.0 × 10^−3^	[47]
*Ni(II)*	N6	DMG	2.0 × 10^−3^	[48]
*Fe(III)*	Zein	curcumin	4.0 × 10^−1^	[49]
*Fe(II)*	PVBC	PIMH	1.0 × 10^−4^ (solution) and 2.0 × 10^−3^ (solid state)	[50]

PAN—polyacrylonitrile, PCDA—10,12-pentacosadiynoic acid, PCDA-5EG—10,12-pentacosadiynoic acid-pentaethylene glycol, PA6—polyamide-6, PVDF—polyvinylidene, Au@GSH NPs—*L*-glutathione-conjugated gold nanoparticles, CA—cellulose acetate, PMDA—pyromellitic dianhydride, PVA—poly(vinyl alcohol), AuNC—gold nanoclusters, PVB—polyvinylbutyral, PANI—polyaniline, PVBC—poly(vinylbenzyl chloride), PIMH—2-(2’-pyridyl)imidazole.
